# Combination of mesenchymal stromal cells and machine perfusion is a novel strategy for organ preservation in solid organ transplantation

**DOI:** 10.1007/s00441-020-03406-3

**Published:** 2021-01-13

**Authors:** Lingfei Zhao, Chenxia Hu, Fei Han, Dajin Chen, Yanhong Ma, Fanghao Cai, Jianghua Chen

**Affiliations:** 1 Kidney Disease Center, the First Affiliated Hospital, College of Medicine, Zhejiang University, Hangzhou, Zhejiang People’s Republic of China; 2 Key Laboratory of Kidney Disease Prevention and Control Technology, Zhejiang University, Hangzhou, Zhejiang Province People’s Republic of China; 3 Institute of Nephrology, Zhejiang University, Hangzhou, Zhejiang People’s Republic of China; 4 State Key Laboratory for Diagnosis and Treatment of Infectious Diseases, the First Affiliated Hospital, College of Medicine, Zhejiang University, Hangzhou, Zhejiang People’s Republic of China

**Keywords:** Mesenchymal stromal cells, Machine perfusion, Organ preservation, Solid organ transplantation, Regenerative medicine

## Abstract

Organ preservation is a prerequisite for an urgent increase in the availability of organs for solid organ transplantation (SOT). An increasing amount of expanded criteria donor (ECD) organs are used clinically. Currently, the paradigm of organ preservation is shifting from simple reduction of cellular metabolic activity to maximal simulation of an ex vivo physiological microenvironment. An ideal organ preservation technique should not only preserve isolated organs but also offer the possibility of rehabilitation and evaluation of organ function prior to transplantation. Based on the fact that mesenchymal stromal cells (MSCs) possess strong regeneration properties, the combination of MSCs with machine perfusion (MP) is expected to be superior to conventional preservation methods. In recent years, several studies have attempted to use this strategy for SOT showing promising outcomes. With better organ function during ex vivo preservation and the potential of utilization of organs previously deemed untransplantable, this strategy is meaningful for patients with organ failure to help overcome organ shortage in the field of SOT.

## Introduction

Solid organ transplantation (SOT) is an optimal, lifesaving treatment choice for patients with life-threatening organ failure. However, there is a considerable imbalance between the demand and supply of donor organs worldwide. For example, kidney transplantation data from the OPTN/SRTR 2018 annual report demonstrated that by the end of the year, the total number of patients on the organ waiting list was nearly 90,000 in the USA, whereas only about 22,000 kidney transplants were conducted in the whole year (Hart et al. 2020). The limited donor pool has become the major obstacle for the widespread clinical application of transplants. Therefore, there is an urgent need to explore more available organs. Transplantation of organs from an expanded criteria donor (ECD) emerges as an alternative in cases where organs from standard criteria donation (SCD) cannot be found. However, compared with organs from SCD, those from ECD are significantly more prone to ischemia/reperfusion (I/R) injury, which is associated with an increased risk of inferior transplant outcomes, including delayed graft function (DGF) and worse long-term graft survival and function (Fellmann et al. 2020; Pascual et al. 2008; Renkens et al. 2005).

Thus, there is a need for adopting novel approaches before transplantation and higher requirements for better evaluation to ensure the quality of the retrieved organs. Organ preservation is an important part of this process. The most widely used donor organ preservation technique is cold static preservation (CSP). Recently developed machine perfusion (MP) has shown its advantages over conventional methods in many aspects (Moers et al. 2009; Tingle et al. 2019). One of these aspects is the possibility of enabling active interventions during preservation (Hosgood et al. 2015). Different interventions, including pharmacologic and/or gene- or cell-based therapies, have been attempted to rehabilitate the viability of grafts with MP. Among them, treatment with mesenchymal stromal cells (MSCs) is a promising strategy.

MSCs are a type of multipotent progenitor cells, which are characterized by a typical spindle shape and the ability to differentiate into chondrocytes, osteoblasts and adipocytes. On their surface, MSCs express high levels of the mesenchymal markers CD105, CD90 and CD73 but do not express the hematopoietic markers CD45, CD34, CD14, CD79a, CD11b and HLA-DR (Dominici et al. 2006). Functionally, MSCs exhibit immune evasive properties accompanied by high immunoregulatory, anti-inflammatory, anti-apoptotic, anti-oxidant and regenerative abilities. Based on these features, MSCs are used in the recovery of several acute diseases, including acute kidney injury, bone repair (Todeschi et al. 2015), acute myocardial infarction (Yannarelli et al. 2013) and acute cerebral infarction (Zong et al. 2017). In the field of SOT, during the last decade, considerable efforts have been channelized towards enabling MSCs to facilitate graft repair. However, despite promising outcomes in animal models, clinical application of MSCs resulted in no remarkable effect (Reinders et al. 2018). One of the major reasons for the disappointing results is the low in situ amounts of infused cells, as it has been reported that the majority of intravenously injected MSCs are trapped in the lungs, liver and spleen (Zhang et al. 2007). Additionally, owing to the harsh microenvironment in vivo after transplantation, the number of surviving MSCs is also rare (Zhang et al. 2018). Moreover, administration of a high concentration of MSCs is not a feasible alternative, as the risk of lethal pulmonary thromboembolism is unbearable (Furlani et al. 2009).

However, the combination of MSCs with MP can effectively overcome the above-mentioned hurdles. With this technique, MSCs can efficiently reach the microvessels of isolated organs, which allows an adequate number of cells to settle in the local site. Meanwhile, ex vivo preservation creates a microenvironment without an allogeneic immune attack, which is in favor of MSC survival and function. These advances enable complete utilization of the potential of MSCs during ex vivo preservation, turning organ preservation from passive storage to active preconditioning and accelerative resuscitation.

In this review, by summarizing the available studies, we intend to provide an integral and updated view of the current understanding of the combination of MSCs with MP for organ preservation. Depending on this strategy, the organs that would previously be discarded may now be reconsidered acceptable for transplantation, which is meaningful for increasing the size of the donor pool and for promoting the therapeutic effect of organ transplant in organ failure patients in the future.

## A brief introduction to organ preservation

Preservation of functionality of isolated grafts is a prerequisite for an effective transplantation. The basic principles of organ preservation are (i) suppression of cellular metabolism to the lowest level minimizing tissue injury and (ii) maximal simulation of the physiologic microenvironment (Salehi et al. 2018).

Organ preservation methods include CSP and MP. CSP is the oldest technique for organ preservation in the transplantation community. Hypothermia can reduce cell metabolic activity and, to a certain extent, minimize graft injury during ex vivo procurement and transport (Bae et al. 2012). Lapchinsky et al. were the first to introduce this method in 1960 in which they concluded that with the help of hypothermic blood, isolated canine kidney could be kept for 24 h and was sufficient for subsequent kidney reimplantation surgery (Lapchinsky 1960). Thereafter, the development of the University of Wisconsin (UW) solution by Belzer et al. initiated the golden age of CSP for human SOT (Wahlberg et al. 1987). Nowadays, apart from UW, multiple other solutions such as the Celsior solution, the Histidine-Tryptophan-Ketoglutarate solution and the Institut Georges Lopez-1 solution (Dondéro et al. 2010; OʼCallaghan et al. 2014; Schneeberger et al. 2009) have demonstrated alternative effects in terms of utility and patient outcomes post-transplantation.

**Fig. 1:**
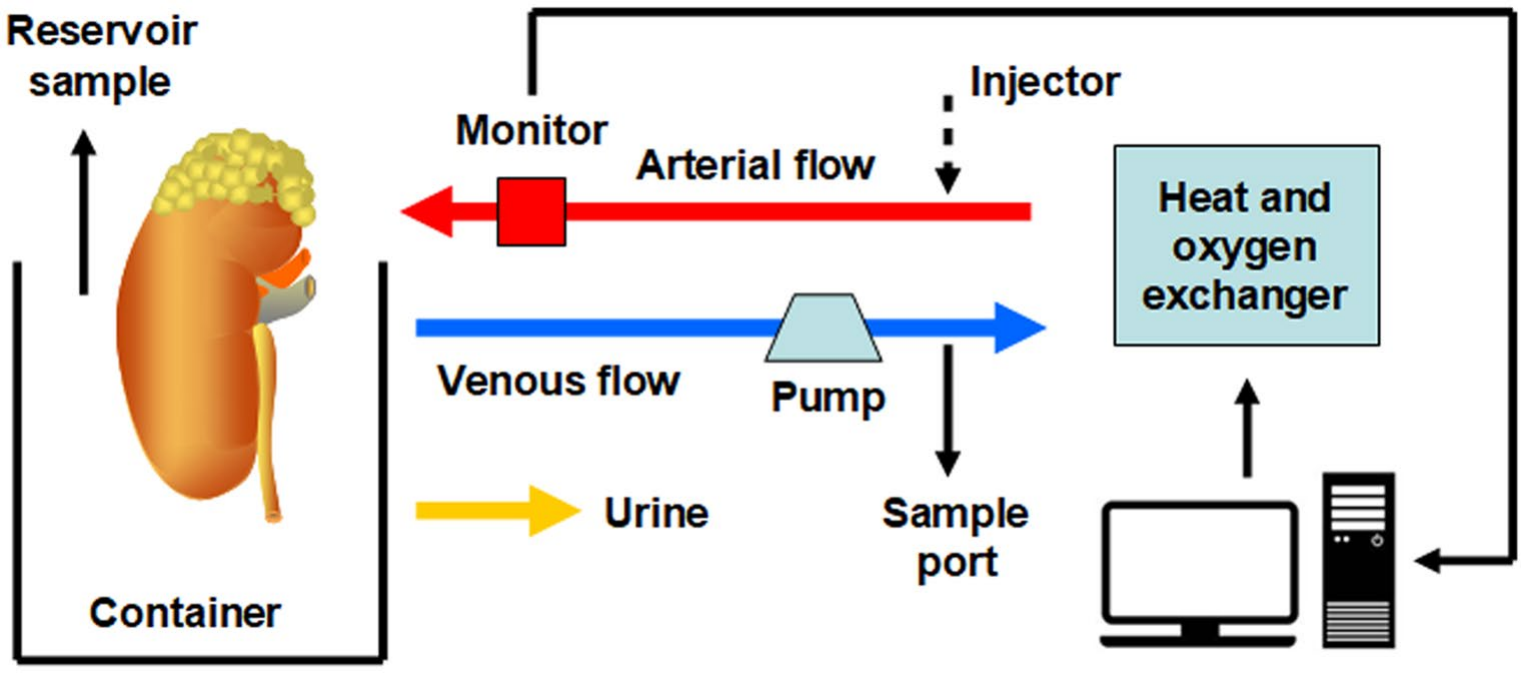
Schematic drawing of the organ preservation MP system (using kidneys as an example). The MP system consists of a pump, circuit, preservation solution, heat and oxygen exchanger and the control system. The solution is pumped into the kidneys through the renal artery, recycled from the reservoir and then is supplied with heat and oxygen in the exchanger, forming a closed loop. The ureter is also cannulated, which permits the timely removal of metabolic waste. Samples can be obtained from the reservoir, circuit and the collected urine. If an injector is provided, exogenous pharmacologic and/or gene- or cell-based therapy can be performed

CSP has been regarded as the standard proposal for most organ preservation in the past decades. However, the increased number of suboptimal graft transplantation cases and the demand for prolonged storage time due to distant geographical areas highlight the limits of CSP (Pirenne 2010). These aspects, together with defects such as cell swelling, acidosis and reactive oxygen species (ROS) production during CSP, have led to the development of MP, as a novel procedure in organ preservation. The MP system consists of a machine pump, preservation solution, circuit, optional temperature control and oxygenation control devices (Fig. 1). Depending on the temperature, this strategy can be divided into hypothermic (HMP, 0–10 °C), subnormothermic (SNMP, approximately 21 °C) and normothermic MP (NMP, 37 °C).

HMP was first started in the 1960s and is one of the most well-developed paradigms. The continuous flow of preservative solution through the vasculature of the donor organ enhances the penetration of the solution within the organ, thus providing better nutrient supplementation. In cases where the solution is oxygenated and renewed, oxygen demand can be fulfilled and metabolic waste can be timely washed out. Under these circumstances, cellular damage is expected to remain at a minimum level and preserved organ function remains closer to the baseline level. Multiple pre-clinical results suggest that this proposal is superior to conventional CSP for the protection of multiple cell structures (Michel et al. 2015; Tozzi et al. 2013). In terms of clinical kidney transplantation, it has become the preferred choice for the preservation of suboptimal kidneys (Gallinat et al. 2012; Wang et al. 2017).

Compared with HMP, SNMP and NMP are thought to be a method of organ preservation that mimics physiological conditions closely. During HMP, oxygenation is always necessary for avoiding ischemia injury, as cellular metabolism is more active at nearly normal temperatures than that in hypothermia. The simulated physiological conditions enable further mitigation of tissue injury and permit a longer preservation time. Brasile et al. investigated the effects of an acellular perfusate for the preservation of canine auto-transplant kidneys during 18 h with warm perfusion at 32 °C (SNMP) compared with HMP. Immediate life-sustaining function after transplantation was observed in the SNMP group, whereas the HMP group did not result in similar restorative effects. This evidence indicates the superior preservation effects of SNMP over HMP (Brasile et al. 2002). Another advantage of SNMP and NMP over CSP is that the normal cellular metabolic activity at these temperatures allows for the continuous evaluation and real-time feedback of graft function prior to transplantation and can provide additional information for selection of transplantable grafts. This is especially useful when considering the transplantation of marginal organs. For example, based on ex situ NMP parameters, such as arterial blood flow, the rate of bile production and ejection fraction, organs that would previously be excluded can now be deemed suitable for transplantation. A small number of clinical cases reported that organs initially declined for transplantation were subsequently transplanted into patients and patients experienced an unremarkable recovery (Hosgood et al. 2016; Watson et al. 2017). The most considerable clinical experience has been observed during lung transplantation, in which oxygenation, hemodynamic and respiratory data and macroscopic appearance serve as key parameters to determine the transplantability of otherwise discarded organs (Wallinder et al. 2012). Finally, based on normal cellular metabolic activity, the near-normothermic microenvironment offers a platform for ex vivo MSC-based therapy. By active preconditioning with these highly regenerative cells, the function of isolated organs can be further improved during ex vivo preservation.

## Theoretical basis of combining mesenchymal stromal cells with machine perfusion as a novel organ preservation strategy

Quality of donor organs is a major factor for rapid function resumption after transplantation and one of the key issues affecting graft quality is I/R injury (Abecassis et al. 2008). I/R injury is an inevitable process in SOT. During donor procurement, once the blood flow is stopped, the oxygen and nutrient supplies rapidly decrease to an extremely low level. To accustom to the sudden alterations in the surrounding microenvironment, cells switch from aerobic to anaerobic respiration. However, the limited energy originating from anaerobic metabolism is insufficient for normal cellular physiological activity, which leads to the disturbance of ion distribution, alteration of membrane potential and cell edema. In the postoperative period, due to improper mitochondrial function, the sudden reperfusion of oxygen induces a local but important production of ROS, which is detrimental to proteins, phospholipids and DNA (Li and Jackson 2002; Raedschelders et al. 2012). Importantly, both hypoxia (ischemia phase) and reoxygenation (reperfusion phase) are deleterious to cellular membranous structures not only for outer but also for inner cellular membranes, including the endoplasmic reticulum, Golgi apparatus, mitochondrial membranes and cytoskeletal microtubules (Kosieradzki and Rowiński 2008). The disruption of cellular membranous structures releases molecules and enzymes, which can be recognized as damage-associated molecular patterns and finally activates the inflammatory response exacerbating the injury (Bon et al. 2012). These processes constitute the basic physiopathological changes during I/R injury, including inflammation, oxidative stress and cell apoptosis. If these changes are not properly addressed, they can cause irreversible damage to the donated organs (Braza et al. 2016; Lin et al. 2014; Rabb et al. 2016).

**Fig. 2:**
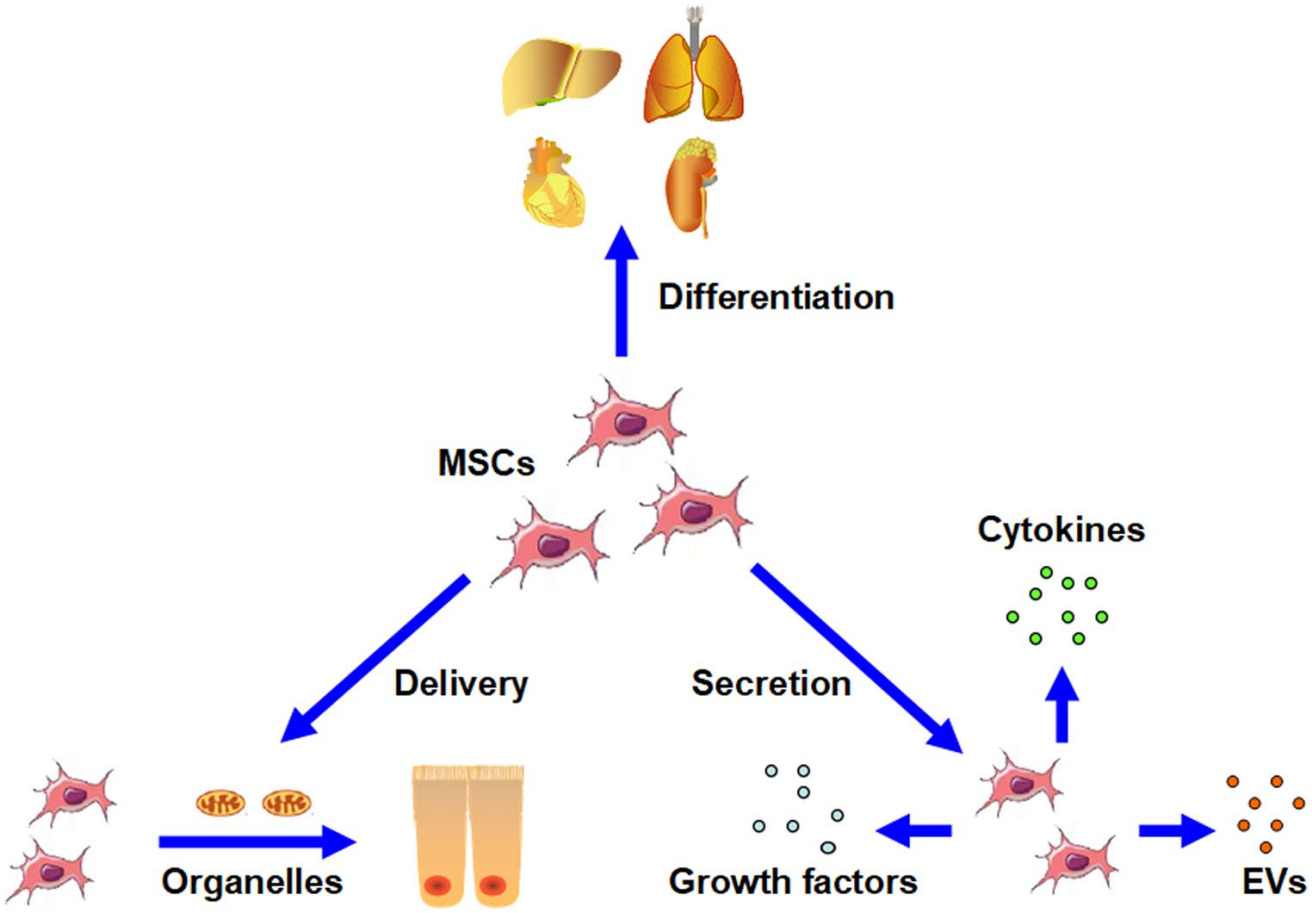
Mechanisms underlying the protective effects of MSCs on I/R-injured organs. Based on multiple actions, MSCs can alleviate the I/R injury on target tissues. First, MSCs can differentiate into related functional cells, thus favoring regeneration. Second, via secretion of various cytokines, growth factors and EVs, MSCs can generate a better post-injury microenvironment. Lastly, MSCs exhibit the ability to directly deliver organelles to injured cells, thereby contributing to survival and proliferation

Strong evidence suggests that MSCs have a potential ability to alleviate I/R injury in various organs, including the heart, kidneys, liver, brain and lungs (He et al. 2019; Li et al. 2015, 2018; Preda et al. 2014; Sadek et al. 2013). This evidence provides a theoretical basis for combining MSCs with MP as a novel organ preservation strategy to improve graft quality in SOT. In the following section, we discuss a few specific mechanisms underlying the protective effects of MSCs on I/R-injured organs (Fig. 2).

Compared with pharmacologic interventions that often have limited target sites, MSCs are believed to relieve this highly complex pathophysiology process through multiple modes of action. First, as MSCs have stem cell properties, they can be engrafted into organs where they can differentiate into related functional cells. The most typical case is that of liver injury. After transplantation by either an intrasplenic or intravenous route, Kuo et al. found that MSCs could engraft into the recipient liver, differentiate into functional hepatocytes and rescue liver function (Kuo et al. 2008). Second, increasing researches support the notion that the generation of a better post-injury microenvironment that favors cell survival, proliferation and dedifferentiation increases the beneficial effects of MSCs in limiting injury and in enhancing regeneration. MSCs can secrete various cytokines and growth factors, presenting paracrine activity. For example, they secrete growth factors, such as brain-derived neurotrophic factor, nerve growth factor and basic fibroblast growth factor, which have been found to be beneficial for reducing neuronal cell apoptosis and for activating endogenous cellular repair programs for recovery in brain ischemia zones (Zhang et al. 2004). Similarly, cytokines such as prostaglandin E2, thrombospondin-2, indoleamine 2,3-dioxygenase and transforming growth factor-β contribute to the balance of the inflammatory response (Meisel et al. 2004; Niu et al. 2017; Németh et al. 2009). Meanwhile, secretion of extracellular vesicles (EVs) is regarded as a special mechanism of paracrine activity presented by MSCs. EVs are heterogeneous vesicles that can be constitutively released by various types of cells, including MSCs. Biologically, EVs are important mediators of cell-to-cell communication. Multiple types of proteins, lipids, mRNAs and miRNAs are carried by EVs. After endocytosis by the target cells, these bioactive substances can modulate cell function (György et al. 2011). In a glycerol-induced renal injury model, the protective effects of MSCs on renal cells correlated with the level of miRNAs in MSCs, thereby indicating a miRNA-dependent mechanism (Collino et al. 2015). By injecting human EVs derived from MSCs (MSC-EVs) into rats suffering from I/R-induced renal injury, Ju et al. observed the presence of human hepatocyte growth factor mRNA and protein in rat tubular cells, thereby facilitating renal cell dedifferentiation and growth (Ju et al. 2015).

Recent studies have indicated that the regenerative capacity of MSCs, especially on the function of some important organelles, may be fulfilled by a direct organelle delivery approach. Compared with modulation by differentiation or paracrine actions that activate downstream effectors, direct organelle delivery is a more efficient and economical strategy. It helps recipient cells expend less time and energy on synthesis of related organelles, which is important in critical situations. Supporting this point, Spees et al. reported that by transferring mitochondria, co-cultured MSCs rescued the fate of lung epithelial cells, which could not survive under standard conditions due to defects in mitochondrial function (Spees et al. 2006).

## Experimental data on combination of mesenchymal stromal cells and machine perfusion in ex vivo resuscitation of donated organs

The evidence mentioned above makes MSCs ideal candidates for ex vivo cell-based therapy in organ preservation. In recent years, the combination of MSCs and MP in ex vivo resuscitation of donated organs was attempted in the kidneys, lungs and liver and some encouraging results were obtained (Table 1).

**Table 1:** Experimental data on the combination of MSCs and MP in ex vivo resuscitation of donated organs

Organ	Year	Model	MSCs source	MP condition	Outcomes	Reference
Kidney	2019	Porcine DCD kidney model	Human AT-MSCs and BM-MSCs	NMP 7 h	NM	Pool et al. (2019)
	2020	Porcine DCD kidney model	Human AT-MSCs and BM-MSCs	NMP 7 h	↓NGAL, LDH; ↑HGF	Pool et al. (2020)
	2017	Rat DCD kidney model	Rat BM-MSCs	HMP 4 h	↑Cell energy metabolism and membrane transport genes; ↓MDA, LDH; ↓Glucose, lactate; ↑Pyruvate; ↓Pathological score	Gregorini et al. (2017)
	2019	Human DCD kidney model	NM	SNMP 24 h	↑ATP; ↑EGF, FGF-2 and TGF-α; ↓Inflammation; ↑PCNA; ↑Mitosis	Brasile et al. (2019)
Lung	2014	Human DBD lung model	Human BM-MSCs	SNMP 4 h	↑AFC	McAuley et al. (2014)
	2017	Mice DCD lung model	Human UC-MSCs	NMP 1 h	↑Lung compliance; ↓PAP; ↓Lung weight; ↓Neutrophil infiltration	Stone et al. (2017)
	2016	Porcine DCD lung model	Human UC-MSCs	NMP 12 h	↑Lung compliance; ↑PaO2/FIO2; ↑VEGF; ↓IL-8	Mordant et al. (2016)
	2015	Human DBD lung model	Human BM-MSC-MVs	SNMP 6 h	↑AFC; ↓Lung weight; ↓Tracheal pressure; ↑Lung compliance; ↓PAP and PVR	Gennai et al. (2015)
Liver	2020	Rat DCD liver model	Rat BM-MSCs	NMP 8 h	↓ALT, AST; ↓Hepatocyte apoptosis; ↓MPO, MDA; ↑GSH; ↓Mitochondrial damage; ↓JNK/NF-κB pathway; ↑AMPK pathway; ↓Pathological score;	Yang et al. (2020a, 2020b)
	2020	Rat DCD liver model	Rat BM-MSCs	NMP 8 h	↓ALT, AST, ALP; ↓Hepatocyte apoptosis; ↓Mitochondrial damage; ↓Macrophage activation; ↓ICAM-1, VCAM-1, vWF; ↓ET-1; ↑eNOS/iNOS; ↓Pathological score;	Yang et al. (2020a, 2020b)
	2019	Rat DCD liver model	Rat BM-MSCs	NMP 4 h	↑Survival rate; ↓ALP, ALT, γ-GGT, TBil; ↓Pathological score	Hou et al. (2019)
	2020	Rat DCD liver	Rat HO-1 modified BM-MSCs	NMP 4 h	↑Survival time; ↓ALP, ALT, AST, γ-GGT; ↓IL-1β, IL-6, and TNF-α; HMGB1; ↓TLR4/NF-κB pathway; ↓Pathological score	Cao et al. (2020)

*MSCs* mesenchymal stromal cells, *MP* machine perfusion, *DCD* donation after circulatory death, *DBD* donation after brain death, *AT-MSCs* adipose tissue-derived MSCs, *BM-MSCs* bone marrow-derived MSCs, *NM* not mentioned, *NGAL* neutrophil gelatinase-associated lipocalin, *HGF* hepatocyte growth factor, *UC-MSCs* umbilical cord-derived MSCs, *BM-MSC-MVs* MVs secreted from BM-MSC, *NMP* normothermic MP, *HMP* hypothermic MP, *SNMP* subnormothermic MP, *MDA* malondialdehyde, *LDH* lactic dehydrogenase, *ATP* adenosine triphosphate, *EGF* epidermal growth factor, *FGF-2* fibroblast growth factor-2, *TGF-α* transforming growth factor-α, *AFC* alveolar fluid clearance, *PAP* pulmonary arterial pressure, *PVR* pulmonary vascular resistance, *VEGF* vascular endothelial growth factor, *ALP* alkaline phosphatase, *ALT* alanine aminotransferase, *AST* aspartate aminotransferase, *MPO* myeloperoxidase, *MDA* malondialdehyde, *GSH* glutathione, *vWF* von Willebrand factor, *VCAM-1* vascular cell adhesion molecule-1, *ICAM-1* intercellular cell adhesion molecule-1, *iNOS* inducible nitric oxide synthetase, *eNOS* endothelial nitric oxide synthetase, *ET-1* endothelin-1, *γ-GGT* γ-glutamyltransferase, *TBil* total bilirubin, *HO-1* heme oxygenase 1, *HMGB1* high-mobility group box 1, *TNF-α* tumor necrosis factor-α

### MSCs in renal recovery

Data from a study by Pool et al. demonstrated that a small proportion of human MSCs could be detected in the lumen of glomerular kidney capillaries after 7 h of NMP with MSCs. Moreover, the retained MSCs were found to be structurally intact, thereby indicating that their potential biological activity could be maintained during perfusion (Pool et al. 2019). In their subsequent study, they found a significantly increased level of hepatocyte growth factor, together with a decrease in neutrophil gelatinase-associated lipocalin (NGAL) and lactic dehydrogenase in the perfusate. This evidence indicates that the infusion of MSCs during NMP can protect the isolated kidney from ischemic damage (Pool et al. 2020).

It is known that impaired energy utilization is a major challenge during organ preservation. To explore the protective role of MSCs combined with MP on energy metabolism of the isolated kidneys during ex vivo preservation, Gregorini et al. conducted a series of experiments. Donated kidneys were first subjected to a 20-min period of warm ischemia and then were preserved with continuous perfusion at 4 °C. In the experimental group, MSC-EVs were added to the HMP. MSCs were isolated from rats that highly expressed enhanced green fluorescence protein (EGFP). In the control group, only HMP was administered. After 4 h of perfusion, the kidneys were retrieved and the effluent fluid was collected for further analysis. EGFP staining showed that MSCs were successfully located in the vessels, tubules and interstitium, without signs of embolism in macrovessels/microvessels. Histologically, renal ischemia damage was ameliorated in kidneys perfused with MSC-EVs compared with that in the control group. At the gene level, the experimental group presented with multiple up-regulated genes, mainly related to cell energy metabolism and membrane transport. The authors also analyzed some markers of ischemia damage in the effluent fluid. The levels of injury markers such as lactic dehydrogenase and lactate were significantly lower in the experimental group than those in the control group, in parallel with the change in malondialdehyde (MDA) levels, which is an oxidative stress indicator. The effluent glucose level was lower in the MSC-EV group, whereas pyruvate levels showed the opposite trend. The authors concluded that via re-balancing of energy metabolism enzymatic machinery, which is vital for cell viability, the addition of MSCs during HMP protected donated organ injury (Gregorini et al. 2017).

Based on the promising experimental results, a more recent study by Brasile et al. was conducted, in which they collected five pairs of human donated kidneys for research. Kidneys in both groups were first flushed using a tissue-engineering platform with warm solution (32 °C). Once oxidative metabolism and vasodilation were adequately restored, MSCs were infused into the renal artery in the experimental group. The total preservation duration was 24 h. Kidneys were then harvested for further analysis. Histological examination revealed no evident fluorescence signal from labeled MSCs in the renal parenchyma. Meanwhile, the total cell count of MSCs in the 24-h perfusate was more than 95% of its initial amount. This evidence suggested that rather than integration into the renal tissue, perfused MSCs remained in the local microcirculatory system. Metabolically, adenosine triphosphate (ATP) concentration showed a significant increase in the MSC-treated kidneys, especially in the medulla S3 segment, where tubules are known to be the most sensitive regions to I/R injury. At the cytokine and growth factor levels, the kidneys that received MSC perfusion presented with a reduced inflammatory status, accompanied with an increased synthesis of several growth factors, including epidermal growth factor, fibroblast growth factor-2 and transforming growth factor-α, compared with those in the control group. These alterations eventually contributed to the upregulation of cell proliferation in donated kidneys, which was confirmed by a higher percentage of proliferating cell nuclear antigen and toluidine blue positive cells, indicators of DNA synthesis and cell mitosis. Altogether, these data mentioned above demonstrate a mechanism that during ex vivo perfusion, in which MSCs demonstrate an important function in the restoration of the metabolic rate, alleviation of inflammation, promotion of mitosis and subsequently favoring injured renal cell recovery (Brasile et al. 2019).

### MSCs in lung function restoration

Lung graft acceptance rate remains the lowest of any SOT. It is estimated that only 15-25% of donated lungs meet the selection criteria and are finally transplanted (Pomfret et al. 2008). In order to increase suitability of lungs for clinical utilization, McAuley et al. collected human lungs from brain-dead donors that had been rejected for transplantation and were preserved in an ex vivo lung perfusion (EVLP) container at 36 °C. Thereafter, they determined the level of alveolar fluid clearance (AFC) in the preserved lung lobes. The AFC level represents the capacity to reabsorb alveolar edema fluid and is associated with good clinical outcomes post-transplantation (Ware et al. 1999). If the AFC level was < 10% per hour, an indicator of poor prognosis, MSCs were administered directly into the perfusate in the experimental group and were continuously perfused for 4 h. The addition of MSCs significantly increased AFC levels in the experimental group compared with those of the control group, which was preserved with perfusion only. Their study proved that the combination of MSCs with SNMP was effective in restoring the function of donor lungs and was able to expand the donor pool by utilizing non transplantable organs (McAuley et al. 2014). However, the former study did not focus on alterations in lung function or overall lung fluid balance. To investigate this aspect, Stone et al. administered human umbilical cord-derived MSCs (UC-MSCs) into the perfusate of a mouse donation after circulatory death (DCD) lung preservation model. Improved lung compliance, pulmonary arterial pressure (PAP), lung weight and neutrophil infiltration were observed in the lungs supplemented with MSCs. This indicated that protection against loss of endothelial barrier integrity and resultant edema during EVLP, MSCs enhanced EVLP-mediated reconditioning of donor lungs (Stone et al. 2017).

To determine the best dosage and route of MSC administration, Mordant et al. conducted a dose escalation study in which MSCs were delivered either through the bronchus or via the pulmonary artery during NMP. Similarly, with an increase in the parenchymal concentration of vascular endothelial growth factor and a decrease in the perfusate concentration of interleukin-8, they demonstrated that intravascular infusion of 5 × 10^6^ MSCs/kg plus NMP showed strong protective effects on lung compliance and PaO_2_/FIO_2_ in pig DCD lungs (Mordant et al. 2016).

Microvesicles (MVs) are a type of EVs, which are 100–1000 nm in size and contain anuclear plasma membrane fragments. Previous studies demonstrated that MVs secreted from MSCs (MSC-MVs) exhibited similar biological activity in vitro and in vivo as MSCs themselves (Biancone et al. 2012; Cantaluppi et al. 2012). Further, MSC-MVs are an excellent alternative to MSCs for clinical application as they circumvent a few intrinsic limitations such as the potential of malignant transformation of MSCs and immunogenicity. In line with the studies mentioned above, results from Gennai et al. suggested that the administration of MSC-MVs contributed to the normalization of AFC level, lung weight and the improvement of some airway and hemodynamic parameters, including tracheal pressure, lung compliance, PAP and pulmonary vascular resistance (PVR) in discarded human donated lungs. Additionally, all the beneficial effects disappeared after exposure to an anti-CD44 neutralizing antibody, thus indicating a CD44-dependent MSC-MV uptake (Gennai et al. 2015).

### MSCs in liver function preservation

Yang et al. explored the combination of MSCs with NMP on DCD liver preservation. Rat liver was subjected to a 30-min period of warm ischemia and then harvested to establish a rat DCD liver model. NMP was performed continuously for 8 h during ex vivo preservation. In the combination group, BM-MSCs were injected via the portal vein immediately after the system was connected, whereas in the control group, only NMP was administered. They found that the alanine aminotransferase (ALT) and aspartate aminotransferase (AST) levels were significantly lower in the combination group, thereby indicating better preservation of liver function. Pathological score showed a parallel trend, especially in hepatocyte apoptosis. Considering that oxidative stress injury is an important aspect of hepatic I/R injury, they analyzed the levels of myeloperoxidase (MPO), MDA and glutathione (GSH) and observed mitochondrial damage in different groups. The combination of MSCs with NMP resulted in the reduction of the levels of MPO and MDA and mitochondrial damage; increased the production of GSH; and ameliorated DCD liver oxidative stress injury. The study data also provided evidence that the normal functioning of the JNK/NF-κB and AMPK pathways was restored after MSC perfusion, both of which are known to be closely related to oxidative stress (Yang et al. 2020a, 2020b). Additionally, regardless of oxidative stress, disorders in the donated liver microcirculatory structure and function are other factors that considerably affect the liver physiological function post-transplantation (Vollmar and Menger 2009). The same group reported that MSCs plus NMP pre-transplantation could also demonstrate this aspect of benefit. By inhibiting macrophage activation, by reducing microvascular leukocyte aggregation, by alleviating endothelial cell injury and by regulating ET-1/NO imbalance, this strategy effectively alleviates hepatic sinusoid congestion, maintains hepatic microcirculation homeostasis and favors the improvement of DCD liver quality (Yang et al. 2020a, 2020b).

All the above-mentioned studies focused only on in vitro alterations and lacked an in vivo analysis. To supplement the related results from in vitro experiments, Hou et al. established a rat DCD liver transplantation model. It is known that biliary epithelial cells (BECs) injury in DCD liver is a major factor for the development of graft dysfunction after transplantation and is related with an extremely high incidence of biliary tract complications (approximately 25–60%) (Desai and Neuberger 2009). To evaluate whether bone marrow–derived MSCs (BM-MSCs) combined with NMP could provide additional protective effects on BECs, they transplanted livers that were perfused with BM-MSCs plus NMP into rats in the experimental group (BM-MSCs + NMP group). Rats in the control group were transplanted with livers preserved only with NPM (NMP group). The duration of the ex vivo perfusion was 4 h. After transplantation, they found that the 14-day survival rate was significantly higher in the BM-MSCs + NMP group than in the NMP group. In terms of liver function, the levels of alkaline phosphatase (ALP), ALT, γ-glutamyltransferase (γ-GGT) and total bilirubin (TBil) recovered to baseline levels in the BM-MSCs + NMP group. NMP reconditioning showed some benefits in the normalization of liver function but could not completely reverse the injury. The pathological score of BECs revealed a change corresponding to that of liver function. The supplementation of BM-MSCs indeed showed its advantages in DCD liver protection (Hou et al. 2019).

Previous studies have demonstrated that preconditioning is an effective method to enhance the therapeutic effects of MSCs in vivo. By preconditioning with cytokines/chemical compounds, thermosensitive hydrogen, or genetic modifications, MSC infusion can overcome some of its major disadvantages in vivo, such as short cell survival time, limited cell function and insufficient colonization rate (Zhao et al. 2019). Cao et al. attempted to perfuse rat DCD liver grafts with BM-MSCs or heme oxygenase-1-modified BM-MSCs (HO-1/BM-MSCs) together with NMP. After 4 h of ex vivo perfusion, the liver was transplanted. Although the BM-MSC group had already demonstrated beneficial effects on survival time, liver function, proinflammatory cytokines, high-mobility group box 1 (HMGB1) expression, TLR4/NF-κB pathway inhibition and morphological changes compared with the control group, the HO-1/BM-MSCs group showed additional benefits (Cao et al. 2020).

## Conclusion and future perspectives

In conclusion, promising experimental data have shown that the novel organ preservation strategy of combining MSCs and MP establishes a bridge between organ procurement and transplantation during which the injury caused by I/R can be substantially alleviated and the graft function can be maintained. This approach creates the possibility of a better planned course of action during the entire perioperative period. Instead of directly considering medical procedures and reducing ischemia time via a hasty decision-making process, doctors can plan and proceed at the ideal moment. We strongly believe that this strategy can pave the way for expanding the number of transplantable grafts and for improving their function, facilitating their acceptance after transplantation, which may revolutionize the practice in the field of SOT in the future.

## Abbreviations

SOT: Solid organ transplantation
ECD: Expanded criteria donor
MSCs: Mesenchymal stromal cells
MP: Machine perfusion
SCD: Standard criteria donation
I/R: Ischemia/reperfusion
DGF: Delayed graft function
CSP: Cold static preservation
UW: University of Wisconsin
ROS: Reactive oxygen species
HMP: Hypothermic MP
SNMP: Subnormothermic MP
NMP: Normothermic MP
EVs: Extracellular vesicles
MSC-EVs: EVs derived from MSC
NGAL: Neutrophil gelatinase-associated lipocalin
MDA: Malondialdehyde
EGFP: Enhanced green fluorescence protein
ATP: Adenosine triphosphate
EGF: Epidermal growth factor
EVLP: Ex vivo lung perfusion
AFC: Alveolar fluid clearance
UC-MSCs: Umbilical cord-derived MSCs
DCD: Donation after circulatory death
PAP: Pulmonary arterial pressure
MVs: Microvesicles
MSC-MVs: MVs secreted from MSC
PVR: Pulmonary vascular resistance
BECs: Biliary epithelial cells
BM-MSCs: Bone marrow-derived MSCs
MPO: Myeloperoxidase
GSH: Glutathione
ALP: Alkaline phosphatase
ALT: Alanine aminotransferase
AST: Aspartate aminotransferase
γ-GGT: γ-Glutamyltransferase
TBil: Total bilirubin
HO-1: Heme oxygenase 1
HMGB1: High-mobility group box 1
